# Investigation of genomic mutations and their association with phenotypic resistance to new and repurposed drugs in *Mycobacterium tuberculosis* complex clinical isolates

**DOI:** 10.1093/jac/dkad252

**Published:** 2023-09-23

**Authors:** Simone Mok, Emma Roycroft, Peter R Flanagan, Johannes Wagener, Margaret M Fitzgibbon

**Affiliations:** Irish Mycobacteria Reference Laboratory, St James’s Hospital, Dublin, Ireland; Department of Clinical Microbiology, School of Medicine, Trinity College Dublin, the University of Dublin, St James’s Hospital Campus, Dublin, Ireland; Irish Mycobacteria Reference Laboratory, St James’s Hospital, Dublin, Ireland; Department of Clinical Microbiology, School of Medicine, Trinity College Dublin, the University of Dublin, St James’s Hospital Campus, Dublin, Ireland; Irish Mycobacteria Reference Laboratory, St James’s Hospital, Dublin, Ireland; Department of Clinical Microbiology, School of Medicine, Trinity College Dublin, the University of Dublin, St James’s Hospital Campus, Dublin, Ireland; Irish Mycobacteria Reference Laboratory, St James’s Hospital, Dublin, Ireland; Department of Clinical Microbiology, School of Medicine, Trinity College Dublin, the University of Dublin, St James’s Hospital Campus, Dublin, Ireland; Irish Mycobacteria Reference Laboratory, St James’s Hospital, Dublin, Ireland; Department of Clinical Microbiology, School of Medicine, Trinity College Dublin, the University of Dublin, St James’s Hospital Campus, Dublin, Ireland

## Abstract

**Background:**

WGS has the potential to detect resistance-associated mutations and guide treatment of MDR TB. However, the knowledge base to confidently interpret mutations associated with the new and repurposed drugs is sparse, and phenotypic drug susceptibility testing is required to detect resistance.

**Methods:**

We screened 900 *Mycobacterium tuberculosis* complex genomes from Ireland, a low TB incidence country, for mutations in 13 candidate genes and assessed their association with phenotypic resistance to bedaquiline, clofazimine, linezolid, delamanid and pretomanid.

**Results:**

We identified a large diversity of mutations in the candidate genes of 195 clinical isolates, with very few isolates associated with phenotypic resistance to bedaquiline (*n* = 4), delamanid (*n* = 4) and pretomanid (*n* = 2). We identified bedaquiline resistance among two drug-susceptible TB isolates that harboured mutations in *Rv0678*. Bedaquiline resistance was also identified in two MDR-TB isolates harbouring Met146Thr in *Rv0678*, which dated back to 2007, prior to the introduction of bedaquiline. High-level delamanid resistance was observed in two isolates with deletions in *ddn*, which were also resistant to pretomanid. Delamanid resistance was detected in two further isolates that harboured mutations in *fbiA*, but did not show cross-resistance to pretomanid. All isolates were susceptible to linezolid and clofazimine, and no mutations found were associated with resistance.

**Conclusions:**

More studies that correlate genotypic and phenotypic drug susceptibility data are needed to increase the knowledge base of mutations associated with resistance, in particular for pretomanid. Overall, this study contributes to the development of future mutation catalogues for *M. tuberculosis* complex isolates

## Introduction

Drug-resistant strains of *Mycobacterium tuberculosis* complex (MTBC) continue to pose a public health threat as 500 000 cases of MDR or rifampicin-resistant TB (RR-TB) were estimated in 2021.^1^ Early diagnosis and effective treatment regimens are required to control the spread and emergence of drug-resistant TB. The WHO consolidated guidelines for treatment of MDR/RR-TB recommend regimens that incorporate new drugs (bedaquiline, delamanid and pretomanid) and repurposed drugs (clofazimine and linezolid).^2^ Based on the WHO grouping of medicines, later-generation fluoroquinolones (moxifloxacin and levofloxacin), bedaquiline and linezolid are classified as group A agents and are prioritized for MDR-TB treatment. Clofazimine is one of the recommended group B agents that can be included. Likewise, delamanid is one of the group C agents that can be included to complete the regimen when agents from group A or B cannot be used. More recently, the WHO published shorter novel regimens for treatment of MDR/RR-TB that comprise bedaquiline, pretomanid and linezolid (BPaL) together, with or without moxifloxacin (BPaLM).^3^

The introduction of the new and repurposed drugs requires capacity for drug susceptibility testing (DST) as it is important to determine the susceptibility profile of MDR-TB isolates, ideally before the commencement of treatment, and to monitor the emergence of drug resistance. However, phenotypic DST of new drugs has been hampered by the limited access to some agents from the manufacturers.^4^ Phenotypic DST for new and repurposed drugs is also limited in Europe.^4^ Furthermore, slow growth of MTBC can cause delayed susceptibility results.^4,5^ WGS is being increasingly used in routine diagnostics and has the ability to identify resistance mutations rapidly.^6–8^ However, the resistance determinants for new and repurposed drugs are less well understood. It is clear that more phenotypic data are required to understand the role of mutations in candidate genes associated with resistance.^9–11^

Bedaquiline is known to inhibit ATP generation in *M. tuberculosis* by targeting subunit C of ATP synthase (encoded by *atpE*).^12^ Likewise, *Rv0678* encodes a transcriptional repressor of the mmpS5-mmpL5 efflux pump, and mutations in *Rv0678* have been associated with increased bedaquiline MICs and cross-resistance to clofazimine.^13^ Other candidate genes implicated in bedaquiline and clofazimine resistance include *pepQ* (encoding a putative cytoplasmic peptidase) and *Rv1979c* (encoding a putative permease).^14,15^ Delamanid and pretomanid are prodrugs that require activation by deazaflavin cofactor F420-dependent nitroreductase (encoded by *ddn*); they disrupt electron transport and inhibit mycolic acid synthesis in *M. tuberculosis.*^16,17^ Similarly, cofactor F420 is synthesized and reactivated by a group of enzymes encoded by *fgd1*, *fbiA*, *fbiB*, *fbiC and fbiD*. Mutations in any of these genes have been described in delamanid and pretomanid resistance.^17^ Linezolid targets the peptidyl transferase binding site of the 50S ribosomal subunit and inhibits translation.^18^ Although linezolid resistance in *M. tuberculosis* clinical isolates is rare, mutations in *rrl* (encoding the 23S rRNA) and *rplC* (encoding the L3 ribosomal protein), both of which cause linezolid resistance, have been described.^19,20^

MTBC strains that are resistant to new and repurposed anti-TB agents are rare to date, especially in low TB incidence countries, like Ireland. Consequently, there are insufficient phenotypic DST data globally to confidently interpret mutations associated with resistance to these agents as evidenced by the current WHO catalogue of mutations for MTBC.^21^ To increase our understanding of the resistance mechanisms, we investigated the frequency of genomic mutations in 13 candidate genes of MTBC clinical isolates and assessed their association with phenotypic resistance to the new and repurposed drugs, independently of their phenotypic drug susceptibility profile. This study will provide a greater insight into the resistance determinants for new and repurposed drugs and contribute to the development of future mutation catalogues for MTBC.

## Materials and methods

### Study design

A total of 900 MTBC isolates, which had WGS data available at the Irish Mycobacteria Reference Laboratory, were included in the study. This genome collection is a representation of MTBC clinical isolates that were recovered between 2001 and 2021 in Ireland, a low TB incidence country. All genomes were investigated by screening for mutations in the candidate genes and their promoter regions associated with resistance to bedaquiline/clofazimine (*atpE*, *Rv0678*, *mmpL5*, *mmpS5*, *Rv1979c*), delamanid/pretomanid (*fgd1*, *ddn*, *fbiA*, *fbiB*, *fbiC*, *Rv2983*) and linezolid (*rrl*, *rplC*). The genomic regions considered in the WGS analysis are listed in Table S1 (available as Supplementary data at *JAC* Online). All SNPs, insertions or deletions in at least one of the candidate genes were considered. Synonymous mutations, previously reported phylogenetic mutations and mutations graded as not associated with resistance in the WHO mutation catalogue for MTBC were excluded from the analysis.^11,21,22^

### WGS

WGS was previously performed using the Illumina MiSeq or MiniSeq platform (Illumina, CA, USA). DNA libraries were prepared using the Nextera XT DNA library preparation kit using a paired-end approach. WGS analysis was performed using the MTBseq pipeline version 1.0.4 for reference mapping, variant calling and lineage identification.^23^ High-frequency variants were identified by one read in both forward and reverse direction, a phred score of at least 20, and if the allele was indicated by a minimum frequency of 75% in mapped reads. TBprofiler version 2.8.11 was also used for genotypic drug resistance prediction.^24^ Cluster analysis was performed by constructing an SNP distance matrix and using a cut-off of 12 SNP differences.^25^ Raw reads were submitted to the European Nucleotide Archive under the project accession number PRJEB60052.

### DST

Phenotypic DST was performed on all MTBC isolates with mutations detected in candidate genes using the BACTEC MGIT 960 system to assess their association with drug resistance. DST was performed using the WHO recommended critical concentrations for bedaquiline (1 mg/L), clofazimine (1 mg/L), delamanid (0.06 mg/L) and linezolid (1 mg/L).^26^ Pretomanid DST was performed on selected isolates using serial 2-fold dilutions from 0.25 mg/L to 2 mg/L, and the provisional EUCAST breakpoint (2 mg/L) was used to determine pretomanid susceptibility.^27^ Delamanid powder was provided by Otsuka Pharmaceuticals (Otsuka, Tokyo, Japan), and bedaquiline fumarate powder was provided by Janssen Pharmaceuticals (Johnson & Johnson, NJ, USA). Likewise, linezolid and clofazimine were obtained from Sigma-Aldrich (St Louis, MO, USA). Pretomanid powder was provided by TB Alliance (Pretoria, South Africa). For phenotypic DST, the inoculum was prepared by using a Day 1 or 2 positive MGIT liquid culture and the testing procedure was performed as previously described.^28^ DST was repeated for resistant isolates, and these were re-tested at higher concentrations for bedaquiline, clofazimine and linezolid (2 mg/L and 4 mg/L), and for delamanid (0.12 mg/L and 0.24 mg/L).

## Results

**Figure 1.**
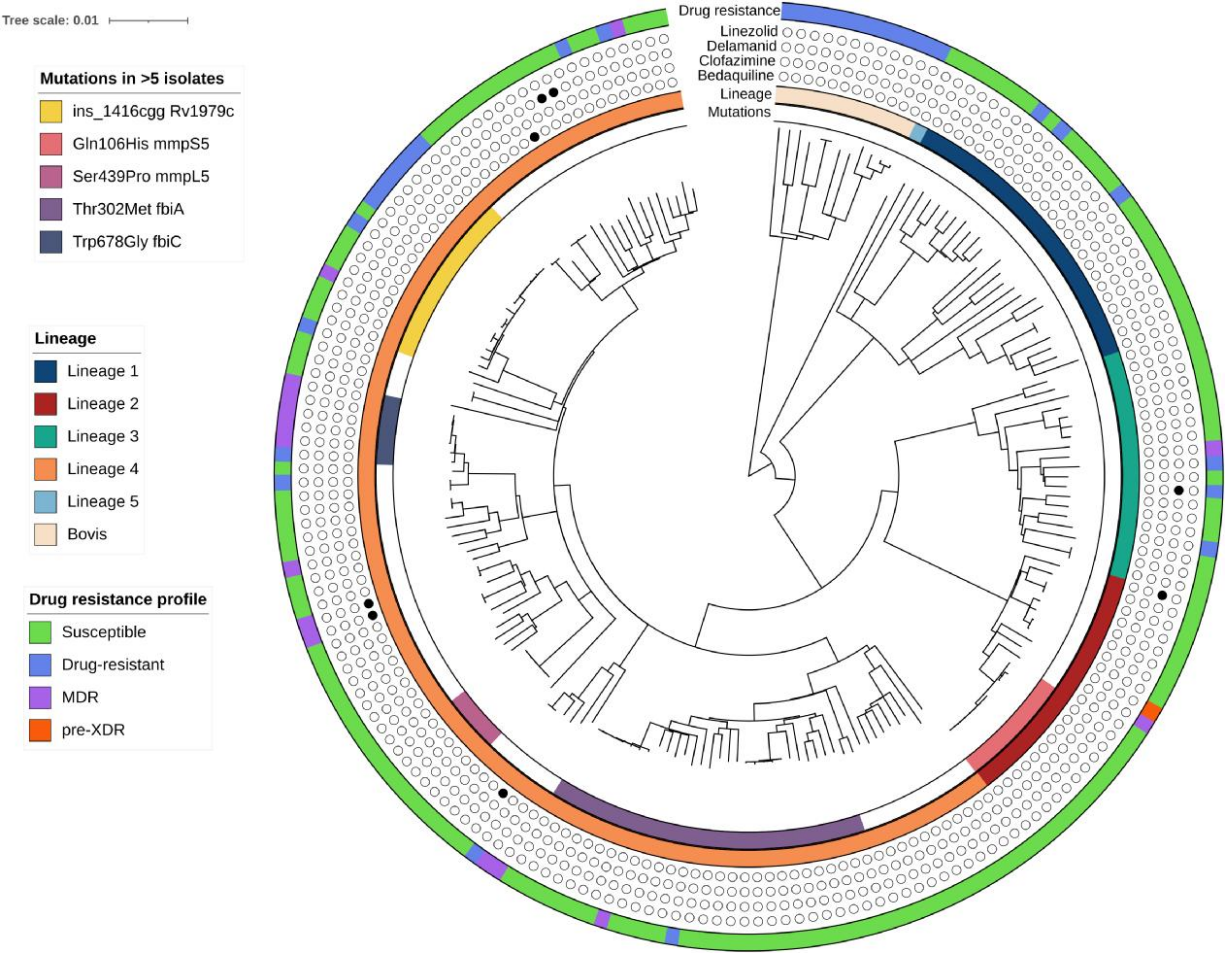
Phylogenetic tree of 195 *M. tuberculosis* complex clinical isolates with mutations identified in candidate genes associated with resistance to new and repurposed drugs. The inner track indicates unique mutations most frequently identified (≥5 isolates) in the candidate genes associated with bedaquiline/clofazimine or delamanid/pretomanid resistance. The second track indicates the lineage of each isolate. Phenotypic drug susceptibility for bedaquiline, clofazimine, delamanid and linezolid is indicated by a circle (filled indicates resistant and unfilled indicates susceptible). The outer track shows the drug resistance profile for each isolate. Details of bedaquiline/clofazimine- and delamanid/pretomanid-resistant isolates are shown in Tables 1 and 2. This figure appears in colour in the online version of *JAC* and in black and white in the print version of *JAC*.

Of the 900 MTBC clinical isolates, 195 were found to have unique mutations in the candidate genes for bedaquiline/clofazimine, delamanid/pretomanid and linezolid. WGS revealed that mutations were detected in five major *M. tuberculosis* lineages, lineage 1 (*n* = 25), lineage 2 (*n* = 21), lineage 3 (*n* = 19), lineage 4 (*n* = 117) and lineage 5 (*n* = 1), and in *M. bovis* strains (*n* = 12) (Figure 1). Genotypic drug resistance profiles of the isolates showed that 147 were drug-susceptible, 32 were resistant to at least one anti-TB drug, 15 were classified as MDR-TB and one was pre-XDR-TB. The most frequently identified mutations in the bedaquiline/clofazimine genes were ins1416cgg in *Rv1979c* (*n* = 15), Gln106His *mmpS5* (*n* = 10) and Ser439Pro *mmpL5* (*n* = 5), which were harboured by isolates within the same clade. Interestingly, Gln106His *mmpS5* and Ser439Pro *mmpL5* were observed only among isolates that had <12 SNP difference and may be cluster-specific mutations. In contrast, ins1416cgg *Rv1979c* and Gln106His *mmpS5* were observed among clustered and unclustered isolates. The most frequently identified mutations in delamanid/pretomanid genes were Thr302Met *fbiA* (*n* = 28) and Trp678Gly *fbiC* (*n* = 6), which were also observed among clustered and unclustered isolates (Figure 1).

### Analysis of mutations detected in bedaquiline and clofazimine candidate genes

Mutations in bedaquiline/clofazimine candidate genes (Ile948Val, Thr794Ile, Asp767Asn in *mmpL5* and a-129g in *Rv1979c*), which were found in >94 isolates, were excluded from analysis as they were already classified in the WHO mutation catalogue as not associated with resistance.^21^ Overall, there were 58 unique mutations detected in bedaquiline/clofazimine candidate genes among 100 MTBC isolates (Table S2). Of the 58 unique mutations, 43% (25/58) were classified as being of uncertain significance and 57% (33/58) were not reported in the WHO mutation catalogue. WGS analysis showed no mutations in *atpE* or the upstream region. There were six mutations identified in *Rv0678* and nine mutations identified in *pepQ*. Likewise, 19 mutations were identified in *mmpL5*, 3 mutations were identified in *mmpS5* and 21 mutations were identified in *Rv1979c*. Among the mutations identified in bedaquiline/clofazimine candidate genes, 66% (38/58) occurred only once. The phenotypic drug susceptibility profile of isolates showed that 77% (77/100) were susceptible to first-line agents, 19% (19/100) were resistant to at least one first-line agent and 4% (4/100) were MDR-TB (Table S3).

Phenotypic DST for bedaquiline and clofazimine was performed on 98 MTBC isolates with mutations identified in candidate genes (two isolates failed to grow and were excluded) (Table S3). There were four confirmed bedaquiline-resistant isolates, which harboured mutations in *Rv0678*. Two isolates harboured Met146Thr, one harboured Ala59Val and one harboured ins-21ttc in *Rv0678*, where all mutations were graded as being of uncertain significance in the WHO mutation catalogue (Table 1). The MIC for bedaquiline-resistant isolates ranged from 2 mg/L to 4 mg/L. There was no association of cross-resistance to clofazimine among bedaquiline-resistant isolates. One bedaquiline-resistant isolate that harboured Ala59Val also had a co-occurring mutation Val344Leu in *mmpL5*. However, three isolates harbouring WT *Rv0678* and Val344Leu in *mmpL5* alone were susceptible to bedaquiline. Of the 94 bedaquiline-susceptible and clofazimine-susceptible isolates, 72 harboured non-synonymous mutations or upstream mutations in bedaquiline/clofazimine candidate genes, and 19 harboured insertions or deletions in *Rv1979c* (Phe144fs, Val351fs, Gly95fs, R473ins) or *mmpL5* (Thr909fs). Likewise, three isolates that harboured nonsense mutations Tyr300* in *mmpL5* and Trp327* in *Rv1979c* were also susceptible to bedaquiline and clofazimine (Table S3). Overall, co-occurring mutations were observed in 11 isolates and 10 were also associated with a susceptible bedaquiline and clofazimine phenotype.

**Table 1.** Overview of genotypic and phenotypic data of bedaquiline (BDQ)-resistant and clofazimine (CLF)-susceptible *M. tuberculosis* clinical isolates

WGS no.	Susceptibility profile	Lineage	Mutation	Gene	BDQ breakpoint^a^ (1 mg/L)	BDQ MIC (mg/L)	CLF breakpoint^a^ (1 mg/L)	CLF MIC (mg/L)	WHO confidence grading of mutation^21^	Other reports of mutation
07IE06	MDR-TB	S-type—4.4.1.1	Met146Thr	*Rv0678*	R	2	S	0.5	Uncertain significance	Observed in 21 MDR-TB strains (L4.4.1.1) with elevated BDQ and CLF MIC by Beckert *et al.*^37^
20IE143a	MDR-TB	S-type—4.4.1.1	Met146Thr	*Rv0678*	R	4	S	0.5	Uncertain significance	
19IE78	DS-TB	Mainly T—4.8	ins-21ttc	*Rv0678* upstream	R	2	S	0.5	Uncertain significance	
21IE14	DS-TB	Ural—4.2.1	Ala59Val, Val344Leu	*Rv0678*, *mmpL5*	R	4	S	1	Uncertain significance	Ala59Val reported by Villellas *et al.* in one bedaquiline-resistant MDR-TB strain (L4)^33^

DS-TB, drug susceptible TB; ins, insertion; R, resistant; S, susceptible.

The WHO recommended critical concentration for BDQ (1 mg/L) and CLF (1 mg/L) was used as the cut-off to determine susceptibility in the BACTEC MGIT 960 system in this table.

### Analysis of mutations detected in delamanid and pretomanid candidate genes

Phylogenetic mutations including a-32g *fbiC*, c-117t *fibC* and Lys270Met *fgd1* were detected in >64 isolates, and Asp113Asn *ddn* observed in lineage 5 was excluded from the analysis. Overall, there were 69 unique mutations identified in the candidate genes associated with delamanid and pretomanid resistance in 114 MTBC isolates (Table S4). Of the 69 unique mutations, 35% (24/69) were classified as uncertain significance and 65% (45/69) were not reported in the WHO mutation catalogue. WGS analysis revealed six mutations in *ddn* and six mutations in *fgd1*. Likewise, there were 20 mutations identified in *fbiA*, 13 mutations in *fbiB*, 19 mutations in *fbiC* and 5 mutations in *Rv2983*. Among the mutations identified 74% (51/69) occurred only once. The phenotypic drug susceptibility profile of the isolates showed 68% (78/114) were susceptible to first-line agents, 21% (24/114) were resistant to at least one first-line agent and 11% (12/114) were classified as MDR-TB (Table S5).

Phenotypic DST for delamanid was performed on 113 isolates with mutations in delamanid/pretomanid candidate genes associated with resistance (one isolate failed to grow and was excluded) (Table S5). There were four confirmed delamanid-resistant isolates, which harboured Tyr29del in *ddn*, Arg31fs in *ddn*, a-239g *fbiA* or Gly8Ala in *fbiA*, where two of the mutations were graded as uncertain significance in the WHO mutation catalogue (Table 2). High-level delamanid resistance was observed in isolates that harboured deletions in *ddn*, with a 4-fold increase in concentration (MIC >0.24 mg/L). The two delamanid-resistant isolates with a-239g *fbiA* and Gly8Ala *fbiA* had an MIC of 0.24 mg/L. Pretomanid DST was performed on four isolates that showed resistance to delamanid; however, only two isolates that harboured deletions in *ddn* had a high pretomanid MIC (>2 mg/L) and were resistant (Table 2). Of the 109 delamanid-susceptible isolates, 104 harboured non-synonymous mutations or upstream mutations in delamanid/pretomanid candidate genes and 5 harboured insertions or deletions in *fbiA* and *fbiC.* Interestingly, co-occurring mutations were observed in 10 isolates that were susceptible to delamanid (Table S5).

**Table 2.** Overview of genotypic and phenotypic data of delamanid (DLM)- and pretomanid (PMD)-resistant *M. tuberculosis* clinical isolates

WGS no.	Susceptibility profile	Lineage	Mutation	Gene	DLM breakpoint^a^ (0.06 mg/L)	DLM MIC (mg/L)	PMD breakpoint^a^ (2 mg/L)	PMD MIC (mg/L)	WHO confidence grading of mutation^21^	Other reports of mutation
19IE114	DS-TB	Beijing—2.2.1	Tyr29del (del_86-88tac)	*ddn*	R	>0.24	R	>2	Uncertain significance	Observed in one L2.2.1 strain reported by Reichmuth *et al.*^35^
19IE77	FLQ-R	Delhi-CAS—3	Arg31fs (del_92 g)	*ddn*	R	>0.24	R	>2	Uncertain significance	
20IE24	DS-TB	Mainly T—4.8	a-239g	*fbiA* upstream	R	0.24	S	≤0.25	Not reported	
20IE133	DS-TB	Mainly T—4.8	Gly8Ala	*fbiA*	R	0.24	S	0.5	Not reported	

del, deletion; DS-TB, drug susceptible TB; FLQ-R, fluoroquinolone resistant; fs, frameshift; R, resistant; S, susceptible.

The WHO recommended critical concentration for DLM (0.06 mg/L) was used as the cut-off to determine susceptibility in the BACTEC MGIT 960 system in this table.^26^ The provisional EUCAST breakpoint for PMD (2 mg/L) was used to determine susceptibility.^27,39^

### Analysis of mutations detected in linezolid candidate genes

WGS analysis showed that no common mutations (G2299T, G2814T, G2270T/C and G2746A) were identified in *rrl* among the dataset. In addition, phenotypic DST for linezolid was performed on a total of 195 isolates and all were confirmed as susceptible. One linezolid-susceptible isolate harboured Pro42Ser in *rplC*, which was not reported in the WHO mutation catalogue, and one linezolid-susceptible isolate harboured Arg38Cys in *rplC*, which was classified in the WHO mutation catalogue as uncertain significance (Table S6).

## Discussion

WGS has been shown to improve TB diagnostics through rapid and accurate detection of resistance mutations. Based on this, phenotypic DST is gradually being replaced by genotypic methods, especially for first-line drugs in low TB incidence countries.^7,8^ Although WGS has the potential to detect resistance determinants to new and repurposed drugs for treatment of *M. tuberculosis*, the molecular mechanisms of resistance are less well understood and phenotypic DST is still required. In 2021, the WHO published the first catalogue of mutations along with a confidence grading for their association with resistance in MTBC, but it includes limited data to interpret mutations identified in candidate genes that are associated with new and repurposed drugs.^21^ In this study, we screened for mutations in candidate genes associated with bedaquiline, clofazimine, linezolid, delamanid and pretomanid resistance in 900 MTBC isolates and found that a large proportion of mutations identified were associated with a susceptible phenotype.

Although resistance to new anti-TB agents is rare, bedaquiline and delamanid resistance is being increasingly reported in MDR/XDR-TB patients whose isolates acquired mutations during treatment and were more likely associated with treatment failure.^29–32^ In addition, bedaquiline and delamanid resistance has been reported in naive *M. tuberculosis* isolates, which suggests that some strains are intrinsically resistant to these drugs.^9,30,33–35^ We also identified two mutations that could be associated with intrinsic resistance to bedaquiline, as two isolates harbouring t-21ttc or Ala59Val in *Rv0678* were susceptible to first-line agents. More recently, bedaquiline-resistant isolates from South Africa have been reported to harbour mutations in *Rv0678*, with mutations at codons 46 to 49 and 67 being the most prevalent.^36^ Although we did not detect mutations in these positions, our results suggest that any mutation identified in *Rv0678* and the upstream region should be investigated to rule out any potential association with reduced susceptibility.

In addition, we identified bedaquiline resistance in two MDR isolates that dated back to 2007, prior to the introduction of bedaquiline. Our WGS analysis showed that it is likely these isolates originated from Eswatini, where a cluster of MDR-TB isolates belonging to lineage 4.4.1.1 has been previously reported with the same mutation (Met146Thr in *Rv0678*) and had elevated MICs to bedaquiline and clofazimine (S. Mok and M. Fitzgibbon, unpublished data).^34,37^ Beckert *et al.*^37^ suggested the mutation Met146Thr *Rv0678* likely emerged years prior to 2009 and before the introduction of bedaquiline treatment in a drug resistance study conducted in Eswatini. It is hypothesized that antifungal azoles may be responsible for the evolutionary pressure in selecting for bedaquiline- or clofazimine-resistant mutations as azole resistance in *M. tuberculosis* has been associated with mutations in *Rv0678*.^34,38^

Similar to a previous study by Reichmuth *et al.*,^35^ we found one isolate belonging to lineage 2.2.1 harbouring Tyr29del in *ddn* that was associated with delamanid and pretomanid resistance. Further analysis of WGS data showed that these isolates were genetically unrelated (95 SNP difference) and suggested that both isolates may have been derived from a common ancestor (S. Mok, unpublished data). Likewise, frameshift (fs) mutation Arg31fs (del_92g) in *ddn* was also associated with delamanid and pretomanid resistance. This suggests that insertions and deletions in *ddn* are likely to be a high confidence marker for delamanid and pretomanid resistance. Bateson *et al.*^39^ also showed that premature stop codons in *ddn* were associated with delamanid and pretomanid resistance. Interestingly, we identified delamanid-resistant isolates that harboured a-239g and Gly8Ala in *fbiA* and had low pretomanid MICs (≤0.25 mg/L). This suggests that there is incomplete cross-resistance between these two drugs and that these mutations might cause delamanid resistance while retaining susceptibility to pretomanid. It is unlikely the strains susceptible to the first-line drugs in this study have been previously exposed to the new drugs, which suggests the presence of strains with intrinsic resistance to bedaquiline and delamanid/pretomanid in Ireland. Although the pre-existing rates of resistance to new and repurposed agents appear to be low, this is a cause of concern as these agents are the last resort for MDR-TB treatment, highlighting the need for accurate phenotypic susceptibility tests to monitor for resistance in clinical isolates prospectively, regardless of phenotypic drug susceptibility profile.

Our study showed that a large proportion of mutations identified in candidate genes were associated with phenotypes susceptible to bedaquiline, clofazimine, delamanid and linezolid. Nonsense and frameshift mutations in *mmpL5* and *Rv1979c* were associated with bedaquiline and clofazimine susceptibility. We identified Trp300* in *mmpL5* in two lineage 1 strains that were susceptible to bedaquiline/clofazimine, and this supports the observations from previous studies that lineage 1.1.1.1 strains harbouring nonsense mutations (or loss-of-function mutations) could be hypersusceptible to bedaquiline and clofazimine.^40,41^ One isolate that harboured a frameshift mutation ins1053g (V351fs) in *Rv1979c* was susceptible to bedaquiline and clofazimine. However, a previous study by Zhang *et al.*^15^ reported the presence of a Val351Ala in *Rv1979c* in a mutant strain of *M. tuberculosis* H37Rv, which was resistant to clofazimine. It was hypothesized that *Rv1979c* encodes a permease that could be involved in protein transport or drug uptake; however, further research is required to determine the effect of mutations at this codon.

Interestingly, we also observed one isolate that harboured Ile208Val in *fbiA*, which has been previously reported in both delamanid-susceptible and -resistant isolates. Reichmuth *et al.* detected the same mutation in one isolate shown to have a low delamanid MIC (≤0.015 mg/L). In contrast, Battaglia *et al.*^9^ reported that Ile208Val was resistant to delamanid and was the most prevalent mutation found in 22 isolates. Further research is required to determine whether the Ile208Val mutation affects the MIC for delamanid.

Linezolid is one of the key agents for treatment of MDR-TB and is part of the novel BPaL and BPaLM regimen.^2^ However, resistance has already been reported in MDR/XDR-TB clinical isolates in high TB burden countries.^19,42–45^ Linezolid resistance in *M. tuberculosis* has been associated with a Cys154Arg mutation in *rplC*, and resistance-associated mutations in *rrl* have also been described. Although we did not detect any mutations associated with linezolid resistance in this study, we identified two linezolid-susceptible isolates that harbour a mutation Pro42Ser or Arg38Cys in *rplC* of uncertain significance.

In conclusion, our study demonstrates that mutations are often present in candidate genes associated with the new and repurposed drugs. However, only a few mutations in *Rv0678*, *ddn* and *fbiA* were associated with phenotypic resistance to bedaquiline, delamanid and pretomanid in this study, whereas clofazimine and linezolid resistance were not found. More studies that correlate sequencing data and phenotypic DST data are required to increase the knowledge base of mutations associated with resistance, in particular for pretomanid. Overall, this study will contribute to the development of future mutation catalogues for MTBC.

## Supplementary Material

dkad252_Supplementary_Data

## Supplementary data

Tables S1 to S6 are available as Supplementary data at *JAC* Online.
